# Type 1 Diabetes eHealth Psychoeducation: Youth Recruitment, Participation, and Satisfaction

**DOI:** 10.2196/jmir.2170

**Published:** 2013-01-29

**Authors:** Robin Whittemore, Sarah S Jaser, Melissa S Faulkner, Kathryn Murphy, Alan Delamater, Margaret Grey, TEENCOPE Research Group

**Affiliations:** ^1^YaleSchool of NursingNew Haven, CTUnited States; ^2^Vanderbilt UniversityDepartment of PediatricsNashville, TNUnited States; ^3^University of ArizonaTucson, AZUnited States; ^4^The Children's Hospital of PhiladelphiaDiabetes Center for ChildrenPhiladelphia, PAUnited States; ^5^University of MiamiMiami, FLUnited States

**Keywords:** Internet, patient participation rates, patient satisfaction, research subject recruitment youth

## Abstract

**Background:**

The Internet and other eHealth technologies offer a platform for improving the dissemination and accessibility of psychoeducational programs for youth with chronic illness. However, little is known about the recruitment process and yield of diverse samples in Internet research with youth who have a chronic illness.

**Objective:**

The purpose of this study was to compare the demographic and clinical characteristics of youth with Type 1 diabetes on recruitment, participation, and satisfaction with 2 eHealth psychoeducational programs.

**Methods:**

Youth with Type 1 diabetes from 4 sites in the United States were invited to participate (N=510) with 320 eligible youth consenting (mean age=12.3, SD 1.1; 55.3% female; 65.2% white; and mean A1C=8.3, SD 1.5). Data for this secondary analysis included demographic information (age, race/ethnicity, and income), depressive symptoms, and recruitment rates, including those who refused at point of contact (22.0%), passive refusers who consented but did not participate (15.3%), and those who enrolled (62.7%). Participation (80% lessons completed) and a satisfaction survey (ie, how helpful, enjoyable) were also analyzed. Chi-square or analysis of variance (ANOVA) analyses were used.

**Results:**

There were significant differences in recruitment rates by income and race/ethnicity such that black, Hispanic, or mixed race/ethnicity and low-income youth were more likely to refuse passively compared to white and higher-income youth who were more likely to enroll (*P<*.001). Participation in program sessions was high, with 78.1% of youth completing at least 4 of 5 sessions. There were no significant differences in participation by program, age, gender, or race/ethnicity. Low-income youth were less likely to participate (*P=*.002). Satisfaction in both programs was also high (3.9 of 5). There were significant gender, race/ethnicity, and income differences, in that girls (*P=*.001), black, Hispanic, or mixed race/ethnicity youth (*P=*.02), and low-income youth (*P=*.02) reported higher satisfaction. There were no differences in satisfaction by program or age.

**Conclusions:**

Results indicate that black, Hispanic, or mixed race/ethnicity youth and low-income youth with Type 1 diabetes are less likely to enroll in Internet-based research than white and higher-income youth; thus, creative recruitment approaches are needed. Low-income youth were less likely to participate, possibly due to access. However, once enrolled, youth of diverse race/ethnicity and low-income youth with Type 1 diabetes were as highly satisfied with the eHealth programs as white youth and those with higher income. Results suggest that eHealth programs have the potential to reach diverse youth and be appealing to them.

## Introduction

Type 1 diabetes is a common chronic illness in adolescents, affecting 1 in 400 youths [1]. The racial/ethnicity distribution of Type 1 diabetes affects primarily white youth in the United States. In a series of studies, the SEARCH for Diabetes in Youth research group reported that the prevalence of Type 1 diabetes in youths is approximately 70% white, 22% Hispanic, and 8% black [2-4]. Management of Type 1 diabetes is complex, requiring frequent monitoring of blood glucose levels, symptoms, and carbohydrate intake. Daily insulin treatment (3-4 injections/day or infusion from a pump) as well as meal-to-meal adjustment of insulin dose depending on diet and activity patterns is required [5]. As youth transition to adolescence and take on greater responsibility for their Type 1 diabetes management and decision making, adherence to diabetes tasks often deteriorates [6], resulting in family conflict, psychological distress, and poor metabolic control [7,8].

Psychoeducational programs for youths and family-based programs have shown to be effective in improving psychosocial and diabetes-related outcomes [9-11]. Psychoeducational programs provide education, behavioral skills, and psychosocial support for young people and their families to learn how to optimally manage a chronic illness. However, disseminating and translating research-based programs into clinical care has been challenging because of provider and family time constraints, as well as cost [12].

The Internet and other eHealth technologies offer a platform for improving the dissemination and accessibility of psychoeducational programs for youth with Type 1 diabetes. Access to the Internet is increasingly available nationwide, with 94% of youth online regularly [13]. Approximately 90% of young people of all demographic and socioeconomic categories have access to the Internet [13]. Thus, eHealth interventions have the potential to reach a diverse group of youths. Programs provided on the Internet can include psychoeducational content, interactive learning, immediate feedback, and social networking [14,15].

Psychoeducational programs delivered via computer-based Internet access have demonstrated efficacy in youths with various chronic illnesses, leading to improved knowledge, symptoms, health outcomes, and quality of life [14,15]. With respect to youths with Type 1 diabetes, an eHealth self-management program with a focus on problem solving and social networking demonstrated improved self-management and problem solving in youth who completed the program compared to a control group [16]. An Internet coping skills training program, developed by our research team, did not demonstrate differential improvements in metabolic control and diabetes-related outcomes compared to an Internet diabetes education program, but youths in both groups reported significantly increased self-care autonomy, higher diabetes self-efficacy, and improved overall quality of life over time [17]. The Internet, therefore, represents a potentially efficient and effective delivery platform for psychoeducational programs for youths with Type 1 diabetes and other chronic illnesses. When evaluated, high satisfaction with eHealth programs have also been reported [18].

Despite the numerous benefits of eHealth programs for youths with chronic illnesses, concerns have been raised about the “digital divide” and Internet access for youths of diverse race and ethnicity and those living in low-income families. Although the vast majority of youths are online, access is higher in white youths and those who live in high-income families [13]. White youths and youths in high-income families are more likely to have online access at home (96%) and go online more frequently compared with black youths (92%), Hispanic youths (87%), and youths from low-income families (86%) [13]. A positive relationship between socioeconomic status and computer-based Internet use has been demonstrated in diverse middle school students [19] and a diverse pediatric clinic population [20].

Challenges of recruiting youths of diverse race and ethnicities for research are well established [21,22]. Issues about the perceived value of research, access to research for families of all strata of society, and cumbersome informed consent procedures have been documented [22]. However, little is known about the recruitment process and yield of diverse samples in Internet research with youths who have a chronic illness.

In addition to concerns regarding access to eHealth research, participation in eHealth programs has varied considerably across studies [14]. For example, in an eHealth program for youth with asthma, participants did not complete self-monitoring on 60% of study days [23]. In an eHealth program for depression, only 30% of youths completed 50% or more of the program modules [24]. In the eHealth problem-solving program for youth with Type 1 diabetes, the mean number of modules completed was 5.22 (of 8), with only 63% of youths completing all modules [18]. Participation in eHealth programs for youths typically decreases over the course of the study [23,25] and higher participation has been associated with more positive outcomes. For example, school-aged youths who had greater participation in an eHealth obesity prevention program demonstrated improved outcomes compared to youths with less participation [26]. A structured environment (ie, school vs home) may improve youth participation in eHealth programs. Youths who participated in a school-based eHealth program for depression had almost a 10-fold higher completion rate for modules and program exercises compared to youths who participated in the same program delivered as open access online [27].

Factors associated with eHealth program participation have begun to be identified. Girls have demonstrated greater participation compared to boys [18,27]. Increased depressive symptoms may also influence participation, although this effect may vary depending on the characteristics of the program, severity of symptoms, and the type of chronic illness. For example, in one study evaluating an eHealth program for depression treatment, less participation was reported in youths with higher depressive symptoms at baseline [24]. In contrast, in another study evaluating an eHealth depression prevention program, the authors reported that youth with higher depressive symptoms had greater participation in the program [28].

In summary, recruitment, participation, and satisfaction with eHealth programs have the potential to influence eHealth program outcomes and generalizability of results. Yet, little research has been undertaken to systematically evaluate the recruitment, participation, and satisfaction of eHealth programs. Therefore, the purpose of this study was to compare the demographic and clinical characteristics of youth with Type 1 diabetes on recruitment, participation, and satisfaction with 2 eHealth psychoeducational programs. Specifically, recruitment, participation, and satisfaction were compared by age, gender, race/ethnicity, household income, metabolic control, and depressive symptoms.

## Method

**Figure 1:**
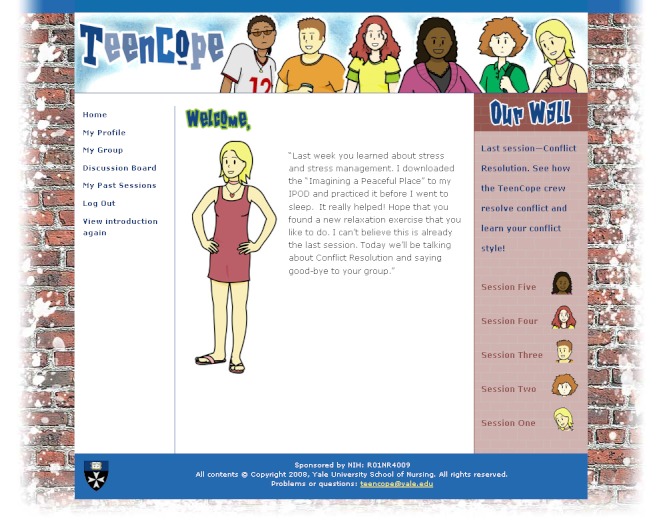
TEENCOPE screenshot displaying home page image, which is updated as teen progresses through the program.

**Figure 2:**
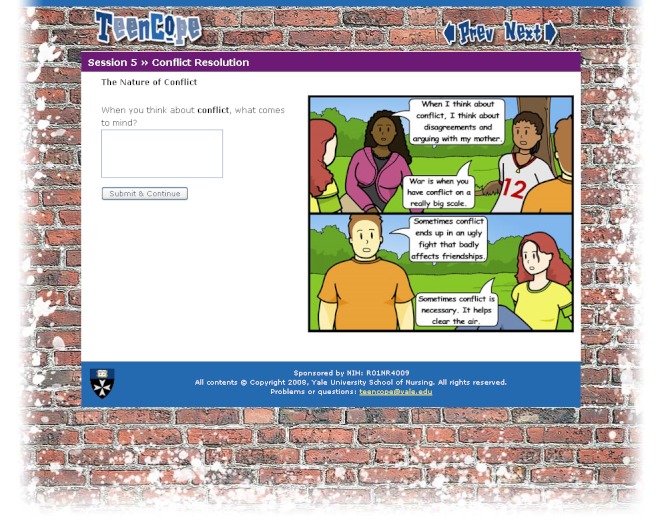
TEENCOPE screenshot displaying graphic novel format for conflict resolution segment.

**Figure 3:**
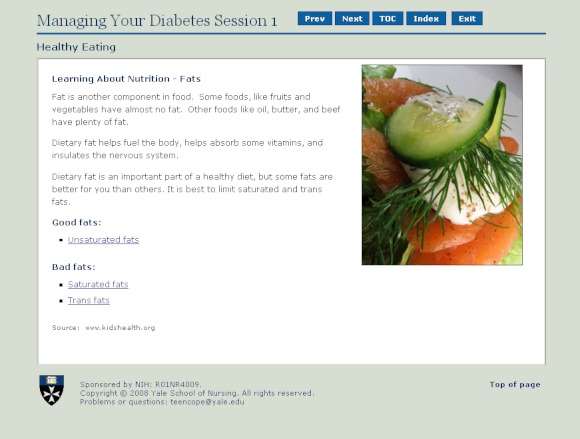
Managing Diabetes screenshot displaying webpage for nutrition segment.

**Figure 4:**
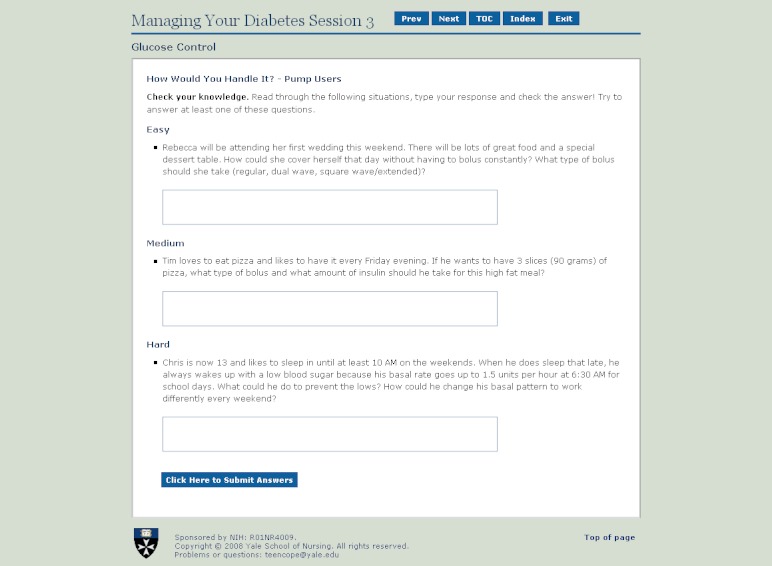
Managing Diabetes screenshot displaying problem-solving questions for glucose control segment.

The current study is a secondary analysis of data from a clinical trial evaluating the effect of an Internet coping skills training program (TEENCOPE) compared to an Internet diabetes health education program (Managing Diabetes) for youth with Type 1 diabetes. Each program consisted of 5 sessions with content tailored to adolescents with Type 1 diabetes. TEENCOPE used a cast of ethnically diverse characters (youth with Type 1 diabetes) and a graphic novel format to model common problematic social situations (ie, parent conflict) and different coping skills to solve the problems (Figures 1 and 2). Managing Diabetes used visuals and a highly interactive interface that allowed adolescents to actively problem-solve diabetes self-management situations (Figure 3 and 4) [29].

A convenience sample was recruited from 4 university-affiliated clinical sites that included Children’s Hospital of Pennsylvania, Philadelphia, PA; University of Arizona, Tucson, AZ; University of Miami, Miami, FL; and Yale University, New Haven, CT. Inclusion criteria were: youth diagnosed with Type 1 diabetes for at least 6 months, aged 11 to 14 years, with no other significant medical problem, school grade appropriate to age within 1 year, able to speak and write English, and access to high-speed Internet at home, school, community, or clinic.

### Procedures

Institutional Review Boards at all clinical sites approved the study. Youth and parents were approached in the clinic setting and informed consent/assent was obtained by trained research personnel. Demographic data were completed by parents at enrollment and email communication was subsequently established with the youth. The youth were sent a link to a password-protected data collection website, and parents were notified of this communication. Internet sites were password protected with all data encrypted and stored on a secure server with hardware and software firewalls. If the youth did not complete online data collection within 1 week, the youth and parents were called approximately 3 times over a 1- to 2-month period as a reminder. Several (2-4) emails and a postcard were also sent during that time in an attempt to re-engage the family. If online data collection was not completed within 3 months, the youth were considered to be passively refusing study participation.

Upon completion of baseline data collection, an automated email was sent to the youths and their parents/guardians to identify their group assignment and provide a link to the appropriate program. (Group assignment was previously determined by randomization of study identification numbers in blocks of 10 to either TEENCOPE or Managing Diabetes.) A unique password was provided to each participant and they were instructed to change this password the first time they logged on to the program. Each program had 5 sessions that were released weekly and took approximately 30 minutes to complete.

A protocol was implemented to enhance participation. Participants were contacted by phone after the first session was released to ensure that they had received the email and were able to access the program. If a youth did not complete a session within 1 week, weekly email, phone, or postcard reminders were sent. Parents were contacted by email if youths did not complete a lesson after 3 weeks. Participants received a gift card (US $25-$30) for completion of online questionnaires evaluating program efficacy.

### Data Collection

Data for this analysis included demographic, recruitment, participation, satisfaction data, and depressive symptoms. Demographic data included gender, age, race/ethnicity, and household income. Recruitment was categorized into 3 groups: (1) refusal at point of contact (refused), (2) those who consented but never established email communication with research personnel or did not complete baseline data (passive refusal), and (3) those who participated (enrolled). Data were not available on income and race/ethnicity for youths who refused at the point of contact. Participation in the Internet programs was categorized into 2 groups: participants who completed the goal of at least 80% of sessions (4 of 5) and participants who completed less than 80% of sessions.

Satisfaction was evaluated with a 6-item survey on how helpful, enjoyable, engaging, easy to use, and worthwhile the program was, as well as how much the skills were practiced. Items were rated on a 5-point Likert scale with higher scores indicative of higher satisfaction (1=not at all; 5=very satisfied). Cronbach alpha in this sample was .73.

Metabolic control was measured with glycosylated hemoglobin (A1C), an estimate of the adolescent’s glycemic control over the past 8 to 12 weeks. The American Diabetes Association (ADA) recommendation for children aged 6 to 12 years is less than 8% [5].

Depressive symptoms were measured with the Children’s Depression Inventory (CDI), a self-report inventory for youth with items on mood, vegetative functions, and interpersonal behaviors [30]. It contains 27 multiple-choice items that yield total scores from 0 to 54. The item that addresses suicidal ideation was eliminated because of the inability to respond immediately to a positive endorsement through the Internet. Higher scores reflect a higher number of symptoms. Youth who scored above the threshold for depression (≥12) were contacted by a qualified study staff member (psychologist, nurse, or social worker) who conducted a depression assessment and made appropriate referrals. Cronbach alpha in our sample was .90.

### Data Analytic Plan

To test for demographic differences in recruitment and participation, Chi-square analyses were conducted to compare categories of recruitment and participation by gender, age, income level, and race/ethnicity. Participation was also compared by metabolic control (A1C within the recommended range, ≤8% above the recommended range) and depressive symptoms (high CDI score ≥12, normal CDI score <12). To test for demographic differences in satisfaction, *t* tests or 1-way analyses of variance (ANOVA) were conducted.

## Results

### Description of Sample

**Figure 5:**
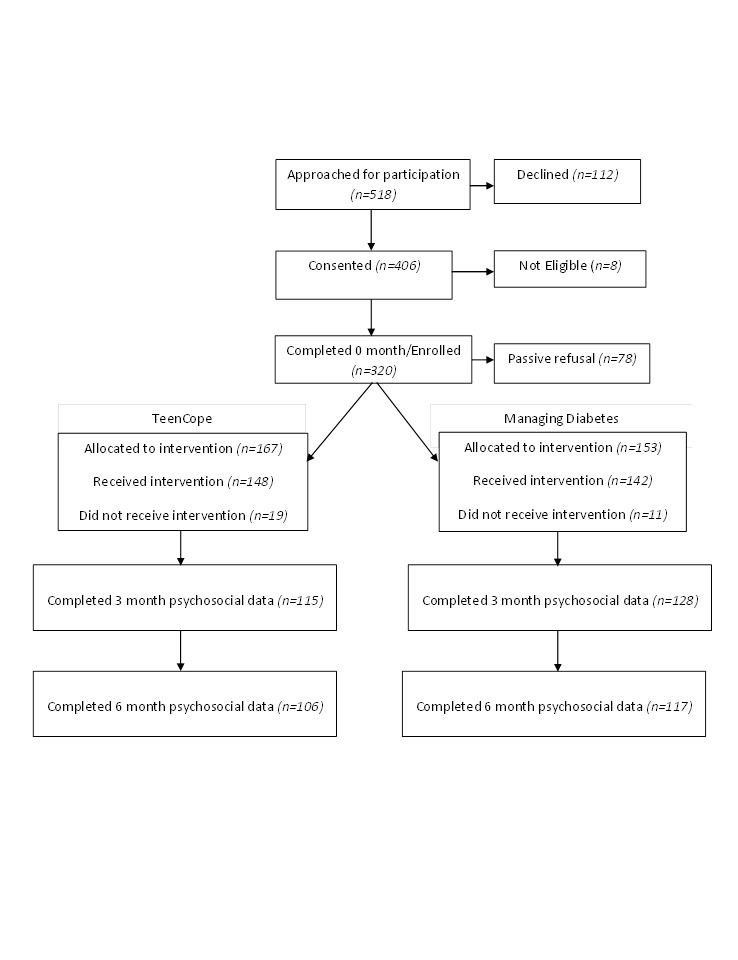
Consort flow diagram.

A total of 518 youth were initially approached to participate in the study. Eight of those teens participated in the study, but were excluded from analysis due to ineligibility. A total of 320 (62.7%) eligible teens enrolled in the study, 112 (22.0%) refused, and 78 (15.3%) passively refused (Figure 5). Reasons for refusal included no interest (n=63, 56.3%), time (n=19, 17.0%), lack of easy access to Internet (n=8, 7.1%), and unknown (n=22, 19.6%).

The mean age was 12.3 years (SD 1.1) and 177 (55.3%) of the sample were female (see Table 1). A total of 204 (65.2%) were non-Hispanic white and 109 (34.8%) were black, Hispanic, or other. Overall, approximately half of the sample (n=165, 52.5%) were above the recommended range for metabolic control (A1C>8%). In terms of depressive symptoms, 53 (16.6%) scored above the clinical cutoff for depression on the CDI (≥12).

**Table 1:** Sample demographics of teen participants (N=320).

Characteristic		Participants
Age (years), mean (SD)		12.3 (1.1)
Duration of diabetes (years), mean (SD)		6.1 (3.5)
**Gender, n (%)**		
	Male	143 (44.7)
	Female	177 (55.3)
**Race/ethnicity, n (%)** ^a^		
	White/non-Hispanic	204 (65.2)
	Black/Hispanic/other	109 (34.8)
**Annual household income (US $), n (%)** ^a^		
	<$40,000	65 (21.0)
	$40,000 - $79,999	87 (28.2)
	>$80,000	157 (50.8)
**Insulin therapy, n (%)** ^a^		
	Pump	189 (59.1)
	Injection (basal)	77 (24.1)
	Injection (conventional)	51 (15.9)
A1C>8%, n (%)^a^		165 (52.5)
CDI≥12, n (%)		53 (16.6)

^a^ The totals for these variables do not equal 320 because some participants chose not to answer these questions or data were not available. Percentages represent valid percent.

Recruitment

Chi-square analyses were used to test for demographic differences by recruitment category (see Table 2). There were no significant differences in recruitment category by gender (χ^2^_2_=4.0, *P*=.14) or age group (11-12 vs 13-14; χ^2^_2_=2.3, *P*=.31). There was, however, a significant difference for race/ethnicity (χ^2^_6_=34.3, *P*<.001) with respect to enrollment. White youth were more likely to enroll and less likely to passively refuse, whereas black, Hispanic, or mixed race/ethnicity youth were less likely to enroll and more likely to passively refuse. There was also a significant difference by income (χ^2^_4_=30.5, *P*<.001), with teens from the lowest income category (annual household income <US $40,000) less likely to enroll and more likely to passively refuse than teens from the higher-income categories (annual household income >US $40,000). It is important to mention that data were not available on race/ethnicity and income for all recruitment categories; data were unavailable on the race/ethnicity and income of youth who refused at the point of contact.

**Table 2:** Demographic differences by for participants who enrolled (n=320), those who refused at point of contact (n=112), and those who refused after consent (n=78) of the total 510 eligible youth who were approached to participate.

Characteristic		Enrolled n (%)	Refused at point of contact^a^ n (%)	Refused after consent n (%)	Chi-square χ^2^ (df)	*P*
**Gender**						
	Male	143 (44.7)	57 (23.8)	40 (16.7)	4.0 (2)	.14
	Female	177 (55.3)	46 (17.6)	38 (14.6)		
	Total^b^	320 (63.9)	103 (20.6)	78 (15.6)		
**Race**						
	Black	25 (8.0)	—	13 ( 16.8)	34.3 (6)	<.001
	Hispanic	59 (18.8)	—	30 (39.0)		
	White	204 (65.2)	—	27 (35.1)		
	Biracial or multiracial	25 (8.0)	—	7 (9.1)		
	Total^b^	313 (80.3)	—	77 (19.7)		
**Household income (US $**)						
	<$40,000	65 (21.0)	—	35 (35.0)	30.5 (4)	<.001
	$40,000-$80,000	87 (28.2)	—	20 (18.7)		
	>$80,000	157 (50.8)	—	18 (10.3)		
	Total^b^	309 (80.9)	—	73 (19.1)		
**Age group**						
	11-12 years	185 (57.8)	53 (49.5)	45 (15.9)	2.3 (2)	.31
	13-14 years	135 (42.2)	54 (50.5)	33 (14.9)		
	Total^b^	320 (63.4)	107 (21.2)	78 (15.4)		

^a^ Data about race/ethnicity and income not available for this group.

^b^ Percentage noted is valid percent, taking into account missing data.

Participation

Participation in the Internet programs was high, with 250 (78.1%) of youth completing at least 4 of 5 sessions, 39 (12.2%) completing 1 to 3 sessions, and 31 (9.7%) completing no sessions. The mean number of sessions completed was 4.08 (SD 1.64) across both groups. There was no significant difference in participation between groups; 129 teens in TEENCOPE participated (completed at least 80% of sessions) at a rate of 77.2%, whereas 121 teens in Managing Diabetes participated at a rate of 79.1% (χ^2^_1_=0.2, *P*=.69).

Results of the Chi-square analyses to test for demographic differences by participation are provided in Table 3. There was no significant difference by gender (χ^2^_1_=1.0, *P*=.31), race/ethnicity (χ^2^_3_=3.1, *P*=.37), or age group (χ^2^_1_=3.1, *P*=.08). There was a significant difference for income (χ^2^_2_=12.6, *P*=.002), with those in the lowest income category (annual household income <US $40,000) less likely to participate, and those in the highest income category (annual household income >US $80,000) most likely to participate. There was no significant difference in participation for metabolic control; adolescents who had an A1C below the recommended cutoff (<8%) were no more likely to participate than those above the cutoff (χ^2^_1_=0.2, *P*=.63). Lastly, depressive symptoms (CDI score) were significantly related to participation (χ^2^_1_=3.9, *P*=.05); youth who scored above the clinical cutoff on depressive symptoms (≥12) were less likely to complete 4 or more sessions than those who scored in the normal range.

**Table 3:** Demographic differences in participation for participators (completed at least 4 sessions or 80%) and nonparticipators (completed <80% of sessions).

Characteristic		Participator n (%)^a^	Nonparticipator n (%)^a^	Chi-square χ^2^ (df)	*P*
**Gender**					
	Male	108 (43.2)	35 (50.0)	1.0 (1)	.31
	Female	142 (56.8)	35 (50.0)		
	Total^b^	250 (78.1)	70 (21.9)		
**Race**					
	Black	20 (8.2)	5 (7.2)	3.1 (3)	.37
	Hispanic	46 (18.9)	13 (18.8)		
	White	162 (66.4)	42 (60.9)		
	Biracial or multiracial	16 (6.6)	9 (13.0)		
	Total^b^	244 (78.0)	69 (22.0)		
**Age group**					
	11-12 years	151 (60.4)	34 (48.6)	3.1 (1)	.08
	13-14 years	99 (39.6)	36 (51.4)		
	Total^b^	250 (78.1)	70 (21.9)		
**Household income (US $)**					
	<$40,000	41 (17.1)	24 (34.8)	12.6 (2)	.002
	$40,000-$80,000	66 (27.4)	21 (30.4)		
	>$80,000	133 (55.4)	24 (34.8)		
	Total^b^	240 (77.7)	69 (22.3)		
**A1C**					
	Recommended (<8%)	118 (48.2	31 (44.9)	0.2 (1)	.63
	High (>12%)	127 (51.8)	38 (55.1)		
	Total^b^	245(78.0)	69 (22.0)		
**CDI**					
	Normal (<12)	214 (85.6)	53 (75.7)	3.9 (1)	.05
	High (>12)	36 (14.4)	17 (24.3)		
	Total^b^	250 (78.1)	70 (21.9)		

^a^ The total number is different by category, due to missing data; some participants chose not to answer questions about race/ethnicity and income.

^b^ Total percentages are valid percent, accounting for missing data.

### Satisfaction

Satisfaction was high with both programs, with no significant difference between groups. The mean satisfaction score was 3.97 (SD 0.71) for TEENCOPE and 3.89 (SD 0.56) for Managing Diabetes. There were significant gender, race/ethnicity, and income differences, in that girls (*t*_202_ *=*3.28, *P=*.001), black, Hispanic, or mixed race/ethnicity (*t*_196_ *=*2.42, *P=*.02), and low-income youth (*F*_2,201_ *=*3.80, *P*=.02) reported higher satisfaction. There was no difference in satisfaction by age or depressive symptoms.

### Summary

Results of the analysis are summarized in Table 4. Lower enrollment rates were demonstrated in youth with Type 1 diabetes who were black, Hispanic, or of mixed race/ethnicity, and of lower income. Lower participation was demonstrated in youth from low-income families. Higher satisfaction was reported by girls, lower-income youth, and black, Hispanic, or mixed race/ethnicity youth.

**Table 4:** Statistically significant comparisons of recruitment category, participation, and satisfaction by demographic characteristics.

Outcome	*P* values			
	Gender	Age	Race/ethnicity	Income
Recruitment/enrollment	—	—	<.001	<.001
Participation	—	—	—	.002
Satisfaction	<.001	—	.02	.02

## Discussion

The 2 psychoeducational eHealth programs for youth with Type 1 diabetes in our study were able to reach a diverse sample, reflective of national prevalence estimates. Overall, there was high participation and satisfaction with the 2 programs. Given the huge influx of eHealth interventions designed for pediatric populations, it is important to understand who they are likely to reach and benefit. Results from the current study highlight demographic differences in recruitment/enrollment, participation, and satisfaction with psychosocial and educational eHealth programs for youth with Type 1 diabetes. Results indicate that black, Hispanic, or mixed race/ethnicity youth with Type 1 diabetes are less likely to enroll in Internet-based research than white youth; thus, creative recruitment approaches are needed. Lower-income youth were also less likely to participate than higher-income youth, possibly due to problems with access. However, once enrolled, black, Hispanic, or mixed race/ethnicity youth and lower-income youth with Type 1 diabetes were as highly satisfied or more satisfied with the eHealth programs as white youth and higher-income youth. These results support the idea that eHealth programs designed for pediatric populations have the potential to reach diverse youth and be appealing to them.

Overall, the rate of enrollment in the study (63%) was average for youth psychoeducational research, which ranges from 49% to 73% [10,21]. The majority of youth who declined participation at the point of contact indicated that they were not interested in the study. As we have shown in a previous study, youth and their families may be less likely to participate in a preventive intervention of this type before the onset of puberty when poorer metabolic control is common [31]. Very few youth indicated that lack of Internet access was a reason for not participating, but they may not have wanted to disclose their lack of Internet access to the clinical or research personnel.

There were no differences in recruitment category with respect to gender or age. There were, however, significant demographic differences in enrollment, with black, Hispanic, or mixed race/ethnicity and lower-income youth more likely to passively refuse, and white youth more likely to enroll. It is possible that black, Hispanic, or mixed race/ethnicity and lower-income youth consent to take part in studies because they feel pressure to please a respected individual (eg, health care providers, research staff) [32], rather than out of a desire to participate. Alternatively, given that black, Hispanic, or mixed race/ethnicity and low-income youth are less likely to be online than white youth [13], youth who passively refused may have had problems with Internet access. In addition, some of the families who had Internet service at the time of enrollment may have later lost or canceled the service because of financial difficulties. These problems with passive refusal (ie, youth who consented to participate but never completed baseline data) are similar to another Internet study with a pediatric population that also was unable to contact some youth after initial consent [33]. Rates of passive refusal in other studies evaluating an eHealth program in youth have ranged from 13% to 40% [18,34-36].

The pattern of results for participation is somewhat different from enrollment; there were no significant differences in race/ethnicity for participation, but there was a significant difference by income. Youth in the lowest income category (family income <US $40,000 year) were significantly less likely to participate than those in the higher-income categories. Again, lower levels of participation may reflect problems with Internet access. Previous studies have shown that lower-income families are less likely to have a home computer and home Internet access [20]. Similarly, lower-income youth report going online less often than higher-income youth; 39% of lower-income teens go online daily vs 75% of higher-income teens [37]. Further, teens from lower-income families are more likely to use the Internet at school, whereas 99% of those from higher-income families access the Internet at home [37]. Thus, it may be less convenient for lower-income teens to participate in eHealth programs than higher-income teens.

Providing options for participation in eHealth programs at schools and clinics may enhance participation. Yet, none of the participants in this study took advantage of Internet access at the clinic for program participation, despite it being offered. Development of eHealth programs for youth in the future may need to use multiple platforms, such as the Internet and smartphones. Currently, 75% of adolescents have cell phones [37]. Latino and black youth are more likely than white youth to access the Internet by cell phone [37]; thus, eHealth programs that are able to be viewed on both the Internet and smartphones may reach more youth of diverse races/ethnicities.

A number of eHealth programs provided on mobile phones with text messaging or smartphone applications have been developed for youth with Type 1 diabetes to enhance blood glucose monitoring [34,38,39] and/or diabetes treatment [40-42]. Improvements in blood glucose monitoring [34] and adherence [41] have been demonstrated. Metabolic control improved only in programs that provided additional components, such as intensive diabetes treatment [41] or behavioral contracting [38]. Studies have primarily been small, 1-group pilot studies, and participation and satisfaction have not been consistently reported. When reported, the reach of the program to youth of diverse races/ethnicities has been low [41], participation has been variable [34,40], and satisfaction has been high [34,40].

Limited literature is available on strategies to promote greater engagement of youth in behavioral interventions, with even less information on the use of the Internet and mobile technologies for minority and low-income youth. Designers of eHealth programs have identified that involving targeted users in the design and development of programs is critical to enhancing user experience and acceptability [43]. It has been proposed that 80% of the impact of an eHealth program is determined during the design phase [44]. Thus, including youth of diverse races and ethnicities in the design and development of eHealth programs appears critical. The use of social media has also been successful in engaging diverse youth in health promotion content by using interactive blogging, connection with others, and creative expression [45]. From the perspectives of African American youth, some evidence supports the value of parental modeling and social support to encourage participation in certain behavioral interventions [46]. Parents of black, Hispanic, or mixed race/ethnicity youth may be key to enlisting their young adolescents’ interest in future eHealth programs. Researchers need to understand parental views on the advantages, safety, and potential health benefits to having their adolescents participate. Novel approaches for obtaining information on parental level of enthusiasm regarding a proposed eHealth investigation through in-person or Skype focus groups could aid in the design of future investigations with minority groups.

Interestingly, our results on satisfaction indicated that black, Hispanic, or mixed race/ethnicity youth and lower-income youth were more satisfied with the program than higher-income and white youth. These high levels of satisfaction are likely a reflection of the inclusion of a culturally diverse “cast” of characters in the program and developmentally appropriate topics and examples. Addressing cultural issues has been shown to foster adherence [47]. Further, we included input from teens at every stage of the program development and paid careful attention to the usability of the program [48]. Finally, we kept text to a minimum, which is more appealing to youth, especially those with low health literacy [49]. Girls were also more satisfied than boys, which may reflect their interest in behavioral topics (ie, interpersonal skills). It has also been hypothesized that girls may respond more to a cognitive-behavioral program based on social learning theory [50].

Finally, there were differences in participation related to depressive symptoms, such that teens who scored above the clinical cutoff were less likely to participate than those who scored below the cutoff. The presence of depressive symptoms may negatively impact motivation for many youth, which has been shown in a previous study of an eHealth smoking cessation program [27]. Youth with higher depressive symptoms may be more likely to participate in programs that they believe will help with their depression [28].

### Limitations

This study provides important new information regarding the role of demographic factors in the study of eHealth programs, but there are several limitations. First, it is important to acknowledge that many youth in our study needed prompts and reminders from research staff to achieve a high level of participation. Further, we did not have data on race/ethnicity and household income for the families who did not enroll in the study. Finally, although the sample was diverse, race/ethnicity and income were highly correlated; only 23% of the high-income youth were not white, making it difficult to determine the relative effect of race/ethnicity and income on participation and satisfaction.

### Directions for Future Research

With the move toward more use of eHealth interventions, it is increasingly important to design programs to maximize recruitment and retention. Although findings from the current study provide important information regarding the demographic factors that are important to consider, future studies are still needed to tease apart the different effects of race/ethnicity and income. Better reporting on recruitment yield (including number approached, ineligible, refused at point of contact, and passively refused) and demographic characteristics of participants and nonparticipants of eHealth programs is needed [51]. In addition, more consistent reporting of youth participation and satisfaction in eHealth research, as well as reasons for refusal are indicated. Recently, a CONSORT eHealth checklist has been published which identifies intended dose and actual dose as well as participant usage over time as important data to report in eHealth clinical trials [52]. Community participatory research strategies to elicit personal perspectives of parents and teens from diverse economic and racial/ethnic backgrounds are needed when designing future eHealth technologies to promote diabetes management. Finally, research on factors that influence youth participation and satisfaction with eHealth programs, including platform for Internet access (ie, home vs smartphone), is also needed.

### Conclusion

As an innovative approach, the use of eHealth programs can improve access to psychoeducational programs for youth of diverse races/ethnicities, socioeconomic status, and with varying chronic illnesses. It is critical that eHealth interventions reach the targeted population to maximize external validity and generalizability of study results. More evaluation of the recruitment, participation, retention, and satisfaction of youth of diverse race/ethnicity and their parents is essential and needed for wider dissemination of future eHealth research.

## Abbreviations

A1C: glycosylated hemoglobin
ADA: American Diabetes Association
CDI: Children’s Depression Inventory

## Multimedia Appendix 1

Consort e-health checklist [52].
